# Metabolic engineering of *Ashbya gossypii* for limonene production from xylose

**DOI:** 10.1186/s13068-022-02176-0

**Published:** 2022-07-15

**Authors:** Gloria Muñoz-Fernández, Rubén Martínez-Buey, José Luis Revuelta, Alberto Jiménez

**Affiliations:** Metabolic Engineering Group, Department of Microbiology and Genetics, Universidad de Salamanca, Campus Miguel de Unamuno, 37007 Salamanca, Spain

**Keywords:** *Ashbya gossypii*, Limonene, Terpenes, Xylose, Metabolic engineering

## Abstract

**Background:**

Limonene is a cyclic monoterpene that has applications in the food, cosmetic, and pharmaceutical industries. The industrial production of limonene and its derivatives through plant extraction presents important drawbacks such as seasonal and climate issues, feedstock limitations, low efficiency and environmental concerns. Consequently, the implementation of efficient and eco-friendly bioprocesses for the production of limonene and other terpenes constitutes an attractive goal for microbial biotechnology. In this context, novel biocatalysts with the ability to produce limonene from alternative carbon sources will help to meet the industrial demands of limonene.

**Results:**

Engineered strains of the industrial fungus *Ashbya gossypii* have been developed to produce limonene from xylose. The limonene synthase (LS) from *Citrus limon* was initially overexpressed together with the native *HMG1* gene (coding for HMG-CoA reductase) to establish a limonene-producing platform from a xylose-utilizing *A. gossypii* strain. In addition, several strategies were designed to increase the production of limonene. Hence, the effect of mutant alleles of *ERG20* (*erg20*^*F95W*^ and *erg20*^*F126W*^) were evaluated together with a synthetic orthogonal pathway using a heterologous neryl diphosphate synthase. The lethality of the *A. gossypii* double mutant *erg20*^*F95W−F126W*^ highlights the indispensability of farnesyl diphosphate for the synthesis of essential sterols. In addition, the utilization of the orthogonal pathway, bypassing the Erg20 activity through neryl diphosphate, triggered a substantial increase in limonene titer (33.6 mg/L), without critically altering the fitness of the engineered strain. Finally, the overexpression of the native *ERG12* gene further enhanced limonene production, which reached 336.4 mg/L after 96 h in flask cultures using xylose as the carbon source.

**Conclusions:**

The microbial production of limonene can be carried out using engineered strains of *A. gossypii* from xylose-based carbon sources. The utilization of a synthetic orthogonal pathway together with the overexpression of *ERG12* is a highly beneficial strategy for the production of limonene in *A. gossypii*. The strains presented in this work constitute a proof of principle for the production of limonene and other terpenes from agro-industrial wastes such as xylose-rich hydrolysates in *A. gossypii*.

**Supplementary Information:**

The online version contains supplementary material available at 10.1186/s13068-022-02176-0.

## Background

Terpenes (terpenoids or isoprenoids) represent one of the largest families of natural compounds with diverse structural and functional features, including mediators of ecological interactions and phytohormones, elements of electron transfer systems, protein modification agents, membrane factors and antioxidants, among others. Terpenes are mostly found in plants, but they also occur in insects, bacteria and fungi [1, 2]. The functional versatility of terpenes allows for an extensive number of industrial applications to be created using this class of natural products such as pharmaceuticals, food additives, pesticides and biofuels [3].

Terpenes are classified according to the number of isoprene (C5) units comprising their structure and include hemiterpenes (C5), monoterpenes (C10), sesquiterpenes (C15), diterpenes (C20), triterpenes (C30) and tetraterpenes (C40) [4]. Limonene is a commonly occurring cyclic monoterpene with a citrus-like aroma, which is found in more than 300 plants and some microorganisms [5]. Limonene and its natural derivatives are widely used as fragrances and flavors, anti-microbials, pesticides, pharmaceuticals, biofuels and biomaterials [5]. Consequently, the market demand for limonene is continuously increasing and the global limonene market is expected to reach 1.9 billion USD by 2024 (https://www.gminsights.com/industry-analysis/dipentene-market). The industrial production of limonene can be carried out through either extraction from plants or chemical synthesis; however, these methods present significant drawbacks regarding seasonal and climate issues, feedstock limitations, low efficiency and environmental concerns [6]. In this regard, different bacterial, yeast and fungi models have been proposed as suitable biocatalysts for the production of limonene and its derivatives [2, 5, 6].

**Fig. 1:**
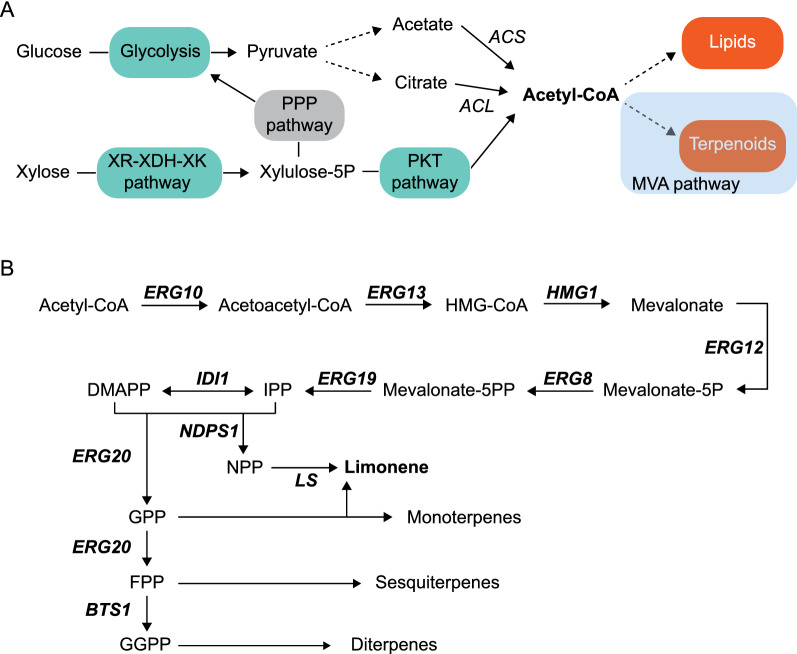
Biosynthesis of terpenes. **A** Schematic pathway for glucose and xylose utilization for the biosynthesis of lipids and terpenes. XR-XDH-XK pathway comprises xylose reductase, xylitol dehydrogenase and xylulose kinase; PKT pathway includes X5P phosphoketolase and phosphotransacetylase. *ACS*, acetyl-CoA synthetase; *ACL*, ATP-citrate lyase. Multi-step pathways are indicated using dashed lines **B** Schematic mevalonate (MVA) pathway. The genes controlling the pathway are in bold

The building blocks of monoterpenes are C5 units of isopentenyl diphosphate (IPP) and dimethylallyl diphosphate (DMAPP), which can be synthesized by either the methylerythritol-4-phosphate (MEP) pathway or the mevalonate (MVA) pathway [2]. While the MEP pathway occurs in most bacteria and plant chloroplasts, the MVA pathway operates in archaea and eukaryotes, including plant cytosol [2]. The eukaryotic MVA pathway initiates with the condensation of two molecules of acetyl-CoA to form acetoacetyl-CoA. Hence, acetyl-CoA is the immediate precursor for both the biosynthesis of terpenes and lipids (Fig. 1A). ﻿Acetoacetyl-CoA is converted to 3-hydroxy-3-methylglutaryl coenzyme A (HMG-CoA), which is transformed into mevalonate by ﻿the enzyme HMG-CoA reductase (encoded by *HMG1*) (Fig. 1B) [3]. The activity of Hmg1 is rate-limiting in the MVA pathway and it is regulated at the transcriptional, translational and post-translational levels [7]. Sequential reactions catalyzed by mevalonate kinase (*ERG12*), phosphomevalonate kinase (*ERG8*), and mevalonate diphosphate decarboxylase (*ERG19*) lead to the conversion of mevalonate into IPP. Then, IPP isomerase (*IDI1*) controls the isomerization of IPP into DMAPP (Fig. 1B) [3] and the condensation of IPP and DMAPP, catalyzed by prenyl transferases, generates prenyl diphosphate molecules of different chain length. Hence, geranyl diphosphate (GPP), which is a precursor of monoterpenes, is synthesized by GPP synthase (*ERG20*). Alternatively, a neryl diphosphate (NPP) synthase (*NDPS1*) can generate NPP, which is the *cis*-isomer of GPP (Fig. 1B). The condensation of additional units of IPP into GPP generates farnesyl diphosphate (FPP; C15) and ﻿geranylgeranyl diphosphate (GGPP; C20), which are the immediate precursors of sesquiterpenes and diterpenes, respectively (Fig. 1B) [4].

*Ashbya gossypii* is a filamentous hemiascomycete that is currently used for the industrial production of riboflavin [8, 9]. In addition, *A. gossypii* has been proposed as an efficient microbial factory for the production of many other metabolites such as nucleosides, folic acid, biolipids, gamma-lactones and recombinant proteins [9–12]. Thereby, *A. gossypii* can be considered as a biotechnological chassis owing to (i) the availability of a large molecular toolbox for systems metabolic engineering, including gene-targeting methods, heterologous expression platforms, or CRISPR/Cas9/Cas12 adapted systems [9, 13–17]; (ii) the ability to grow using low-cost oils and industrial residues such as lignocellulosic hydrolysates, molasses, and crude glycerol [8, 18, 19]; and (iii) and the ease of mycelial harvesting by simple filtration [11], thus making the *A. gossypii* bioprocessing cost-effective.

Recently, engineered strains of *A. gossypii* were shown to produce high levels of biolipids using xylose as the principal carbon source [18, 20]. The overexpression of the native xylose-utilizing pathway (xylose reductase-xylitol dehydrogenase-xylulose kinase; XR-XDH-XK), together with the overexpression of an heterologous phosphoketolase (PKT) pathway enabled channeling metabolic flux from xylose to acetyl-CoA and biolipids (Fig. 1A) [20]. Therefore, both the production of biolipids and terpenes, which share acetyl-CoA as the basic precursor, can be approached using similar strategies. Indeed, it has been reported that xylose utilization can enhance the synthesis of acetyl-CoA derived products such as terpenes as compared to glucose utilization [21]. Also, coupling an heterologous PKT pathway to the biosynthesis of β-farnesene contributes to the increase of cytosolic acetyl-CoA levels with a reduced energy requirement, reduced carbon loss, and improved redox balance [22]. Different terpenes with industrial relevance such as squalene, amorphadiene, β-carotene, limonene, and 1,8‑cineole have been produced in *Saccharomyces cerevisiae* or *Rhodosporidium toruloides* from xylose-based cultured media [21, 23, 24].

The development of efficient biocatalysts for the production of terpenes has involved different metabolic engineering strategies. The overexpression of a truncated form of Hmg1 (tHmg1) solely comprising the catalytic domain is generally recognized to be beneficial for terpene production [7], although several works have shown that the overexpression of the native full-length Hmg1 can provide better results for the production of limonene, linalool and β-carotene [25–28]. Enhancing GPP/NPP synthesis is another general strategy for increasing the yield of limonene and other monoterpenes. In this regard, the biosynthesis of monoterpenes is hindered by the activity of Erg20, which is a bifunctional enzyme that catalyzes two successive steps of the pathway (Fig. 1B). Hence, different approaches have been shown to increase the GPP/FPP ratio: (i) protein engineering of Erg20 to generate mutants with higher GPP synthase and lower FPP synthase activities [6, 29, 30]; (ii) fusion of the Erg20 mutants with monoterpene synthases [29, 31]; and (iii) the dynamic regulation of Erg20 activity by using either inducible promoters or degron tagging [32, 33]. The utilization of a synthetic orthogonal pathway based on NPP, instead of GPP, for the production of monoterpenes has been also shown to increase the production of limonene. The orthogonal pathway employs an NPP synthase and does not require GPP, bypassing Erg20, which connects GPP to the production of other terpenes and the basic metabolism [32, 34]. The overexpression of other genes from the mevalonate pathway such as *ERG12*, *ERG8* and *IDI1* has been also reported to increase the production of terpenes such as α-pinene, limonene, linalool and α-santalene [27, 28, 35, 36].

The current study presents the development of novel *A. gossypii* strains that have been extensively engineered for the production of limonene using xylose as the carbon source.

The native xylose-utilizing pathway was combined with an heterologous PKT pathway to provide a larger acetyl-CoA cytosolic pool. In addition, the mevalonate pathway was engineered to channel acetyl-CoA flux toward limonene production using an orthogonal approach. In sum, we report a novel microbial biocatalyst that can be useful for the production of terpenes from xylose-rich biomass.

## Results

### Implementation of a limonene production system from xylose in *A. gossypii*

Our first strategy to produce limonene in *A. gossypii* using xylose as the only carbon source involved the heterologous expression of a limonene synthase (LS), together with the overexpression of the native Hmg1, in *A. gossypii* strains that can utilize xylose. These xylose-utilizing strains were previously described and contain overexpression modules both for the native xylose-utilizing pathway (XR-XDH-XK), and for an heterologous PKT pathway (Fig. 1A) [20].

**Fig. 2:**
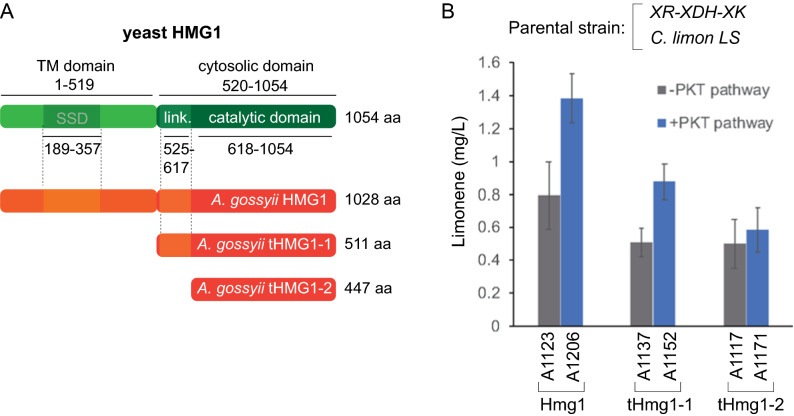
Analysis of the overexpression of Hmg1 isoforms for limonene production in *A. gossypii*. **A** Domain organization of the yeast Hmg1 protein (green); the *A. gossypii* isoforms (Hmg1, tHmg1-1 and tHmg1-2) are depicted (orange) for comparison with the functional domains of the yeast protein. **B** Limonene production of the *A. gossypii* engineered strains with different HMG1 isoforms and either containing or lacking the PKT pathway. The parental strain contained overexpression modules for the XR-XDH-XK pathway and the *C. limon LS*. The results are the means of two independent experiments performed in duplicate. The cultures were performed in flasks for 72 h with 0.5% glucose plus 2% xylose as the carbon sources

Two different limonene synthases have been described from *Citrus limon* (ClLS1 and ClLS2). While CltLS1 shows high (99%) selectivity for the production of limonene and can be functionally expressed in different microbial chassis after removal of the plastid targeting signal [37], CltLS2 was reported to be inactive in *R. toruloides* [38]. Hence, a truncated codon-optimized form of the *C. limon LS1* (*tLS*) (Additional file 1) was synthesized and heterologously overexpressed in *A. gossypii* under the control of the strong promoter *P*_GPD1_ [15]. In addition, the overexpression of three different isoforms of the native Hmg1 was evaluated. The yeast Hmg1 (1054 aa) comprises a transmembrane (TM) domain (residues 1–519) and a cytosolic domain (residues 520–1054). The catalytic domain is located in the cytosolic segment of the protein separated by a linker from the sterol-sensing domain (SSD), which is found in the TM domain (Fig. 2A). Accordingly, we chose to analyze the performance of three different isoforms of the *A. gossypii* Hmg1: a full-length isoform comprising 1028 aa; a truncated isoform lacking the putative TM domain (tHmg1-1, 511 aa); and a truncated isoform excluding both the linker and the TM domain (tHmg1-2, 447 aa) (Fig. 2A). Consequently, each of the Hmg1 isoforms was simultaneously overexpressed, using the strong promoter *P*_GPD1_, with the *C. limon* tLS in xylose-utilizing strains either lacking or containing the PKT pathway. Our results showed that the *C. limon* tLS is functional in *A. gossypii* and can catalyze the synthesis of limonene (Fig. 2B). In addition, the utilization of the full-length isoform of Hmg1 showed superior results for the production of limonene, compared with the truncated isoforms. The presence of the PKT pathway, which favors the channeling of xylulose-5P towards the synthesis of acetyl-CoA, significantly improved the production of limonene from xylose as the carbon source in all of the strains tested, reaching about 1.4 mg/L in the strain that overexpressed the wild-type Hmg1 (Fig. 2B; Table 1). Other terpenes, such as α-pinene and sabinene were not identified in the modified strains, thereby confirming the high selectivity of the *C. limon tLS*.

**Table 1:** Limonene titer of selected engineered strains on *A. gossypii*

Strain number	Modifications^a^	Limonene titer (mg/L)	Limonene titer (mg/g)
A1206	*XR-XDH-XH* + *PKT* + *tLS* + ***HMG1***	1.38 ± 0.15	0.15 ± 0.01
A1224	*XR-XDH-XH* + *PKT* + *tLS* + *HMG1* + ***IDI1***	2.27 ± 0.16	0.33 ± 0.06
A1242	*XR-XDH-XH* + *PKT* + *tLS* + *HMG1* + ***ERG20***	0.17 ± 0.2	0.03 ± 0.03
A1304	*XR-XDH-XH* + *PKT* + *tLS* + *HMG1* + ***erg20***^***F95W***^	2.9 ± 0.25	0.45 ± 0.01
A1251	*XR-XDH-XH* + *PKT* + *tLS* + *HMG1* + ***erg20***^***N126W***^	2.34 ± 0.13	0.43 ± 0.01
A1238	*XR-XDH-XH* + *PKT* + *tLS* + *HMG1* + ***tNDPS1***	33.6 ± 7.8	2.6 ± 1.17
A1308	*XR-XDH-XH* + *PKT* + *tLS* + *HMG1* + *tNDPS1* + ***ERG12* (72 h)**	173.33 ± 18.49	36.89 ± 4.05
A1372	*XR-XDH-XH* + *PKT* + *tLS* + *HMG1* + *tNDPS1* + *ERG12* + ***erg20***^***F95W***^	96 ± 15.98	19.55 ± 3.25
A1388	*XR-XDH-XH* + *PKT* + *tLS* + *HMG1* + *tNDPS1* + *ERG12* + ***erg20***^***F95W***^** (no overexpression)**	143.3 ± 8.76	60.72 ± 3.71
A1308	*XR-XDH-XH* + *PKT* + *tLS* + *HMG1* + *tNDPS1* + ***ERG12* (96 h)**	336.4 ± 13.86	81.88 ± 3.93

^a^Bold indicates the gene that was overexpressed/manipulated in each engineered strain

### Manipulation of IPP utilization for the optimization of limonene production

**Fig. 3:**
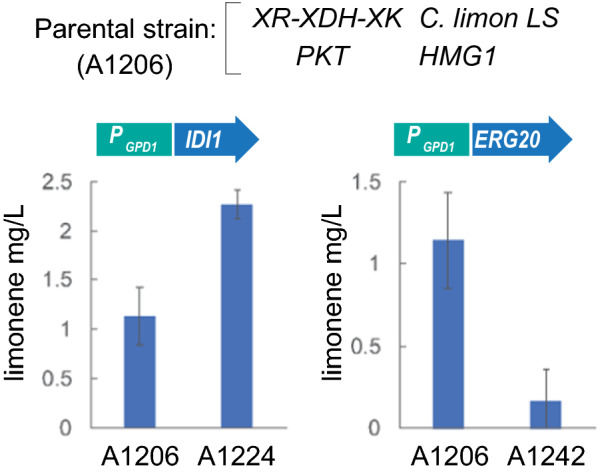
IPP utilization is critical for the production of limonene. The native genes *IDI1* and *ERG20* were overexpressed in the parental strain A1206. The production of limonene was measured in flask cultures grown for 72 h with 0.5% glucose plus 2% xylose as the carbon sources. The results are the means of two independent experiments performed in duplicate

Both IPP and DMAPP are basic building blocks for the synthesis of limonene. Accordingly, we decided to analyze the effect of the manipulation of genes controlling IPP metabolism over the production of limonene in *A. gossypii*. The parental strain used to evaluate these modifications comprised the overexpression of the xylose-utilizing pathway, the PKT pathway, the wild-type Hmg1 and the *C. limon* tLS (Fig. 2B). Hence, the endogenous gene *IDI1* coding for IPP isomerase was overexpressed in the parental strain, using the strong promoter *P*_GPD1_, leading to a significant increase in the production of limonene from xylose-based culture media, which reached 2.3 mg/L (Fig. 3; Table 1). In contrast, the overexpression of the native *ERG20*, which utilizes IPP/DMAPP and controls both the synthesis of GPP and FPP, resulted in a marked decrease in the production of limonene, compared with the parental strain (Fig. 3; Table 1). These results indicate that engineering the utilization of IPP/DMAPP can be critical for the optimization of limonene production. In this regard, strategies intended to generate a higher GPP/FPP ratio would favor the production of limonene.

### Engineering *ERG20* to increase the GPP flux towards limonene production

**Fig. 4:**
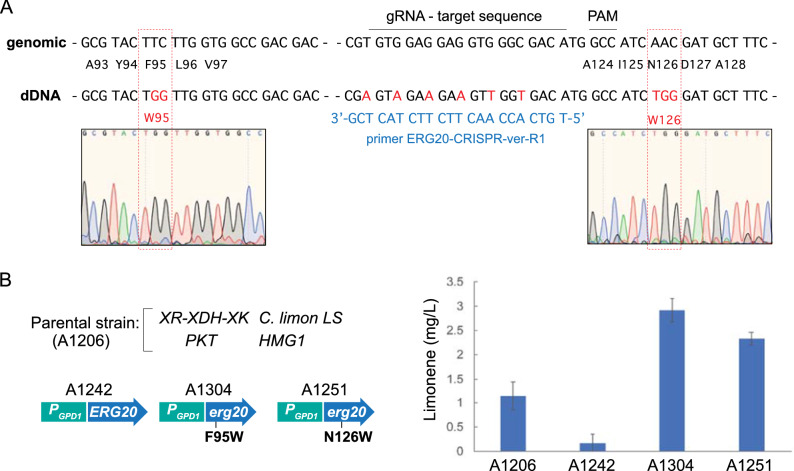
CRISPR/Cas9 genomic edition of *ERG20*. **A** A synthetic sgRNA-dDNA fragment was designed to introduce the selected nucleotide substitutions (red). The *ERG20* genomic locus is shown and both the target sequence and the PAM sequences are indicated. The donor DNA (dDNA) is shown with the designed mutations. The sequences corresponding to the F95W and N126W amino acid changes are highlighted in red. The sequence of the primer used for the analytical PCR is shown in blue. **B** The effect of the overexpression of the native *ERG20* gene and the mutant alleles *erg20*^*F95W*^ and *erg20*^*N126W*^ were evaluated in the parental strain A1206. The production of limonene in the engineered strains was measured in flask cultures grown for 72 h with 0.5% glucose plus 2% xylose as the carbon sources. The results are the means of two independent experiments performed in duplicate

Erg20 is a bifunctional enzyme catalyzing the successive biosynthesis of GPP and FPP. Previous studies have shown that the utilization of mutant alleles of *ERG20* can reduce the FPP synthase activity of the enzyme, thereby contributing to enhancing the production of monoterpenes by increasing the GPP/FPP ratio. In particular, the yeast double mutant *erg20*^*F96W−N127W*^ showed a tenfold increase in monoterpene synthesis without drastically affecting FPP production, which is essential for sterol synthesis [29]. The *A. gossypii* Erg20 contains conserved amino acids (F95 and N126) at the corresponding positions of the yeast protein (F96 and N127) (Additional file 2). Therefore, we sought to analyze in *A. gossypii* the effect of the overexpression of single and double mutants of the native *ERG20* gene. Therefore, a specific CRISPR/Cas9 module (Additional file 1) was designed to introduce the substitutions F95W and N126W in the Erg20 protein of *A. gossypii* (Fig. 4A). While heterokaryotic clones harboring both F95W and N126W substitutions were easily obtained (Additional file 3), the sporulation of these primary heterokaryons did not produce homokaryotic clones harboring the mutant nuclei, which in turn indicated that the *erg20*^*F95W/N126W*^ allele was lethal in *A. gossypii*. In contrast, single mutant (*erg20*^*F95W*^ and *erg20*^*N126W*^) homokaryons were readily obtained, and the presence of the designed substitutions was confirmed by DNA sequencing (Fig. 4A). We also used the single mutants to introduce the corresponding secondary substitutions, but again we were only able to detect both mutations in heterokaryons. The production of limonene was analyzed in the strains overexpressing the *erg20* mutant alleles by comparing it with both the overexpression of the wild-type *ERG20* and the parental strain. Our results showed that the presence of either the F95W or the N126W substitution significantly enhanced (two to threefold higher) the production of limonene with respect to the parental strain (Fig. 4B; Table 1). In particular, the F95W mutation exhibited better performance than N126W, reaching 2.9 mg/L of limonene in xylose-based culture media.

### Bypassing Erg20 through NPP synthesis for the production of limonene

**Fig. 5:**
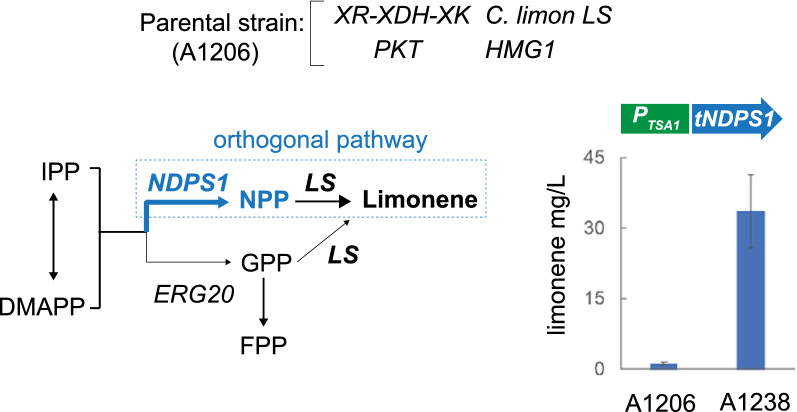
Synthetic orthogonal pathway for the production of limonene in *A. gossypii*. The overexpression of the *tNDPS1* gene from *S. lycopersicum* creates a metabolic bypass of *ERG20* (orthogonal pathway). The limonene synthase (LS) can utilize both NPP and GPP. The *tNDPS1* gene was overexpressed using the strong promoter *P*_TSA1_ in the parental strain A1206. The production of limonene in the engineered strain was measured in flask cultures grown for 72 h with 0.5% glucose plus 2% xylose as the carbon sources. The results are the means of two independent experiments performed in duplicate

A different approach for modifying the utilization of IPP/DMAPP is the implementation of a synthetic bypass of Erg20 using an heterologous NPP synthase and, thus, conferring orthogonality to the synthesis of limonene since NPP cannot be transformed into FPP (Fig. 5). The synthetic pathway can channel metabolic flux towards limonene production through NPP, and its activity is decoupled from the GPP/FPP ratio. Consequently, a truncated and codon-optimized form of the *NDPS1* gene from *Solanum lycopersicum* was synthesized (Additional file 1). The *tNDPS1* gene was assembled in an integrative expression module and was heterologously overexpressed in the parental strain under the control of the strong promoter *P*_TSA1_, to avoid genomic instability due to the recurrent utilization of *P*_GPD1_ [15]. The newly engineered strain was cultured in xylose-based media and produced a limonene titer approximately 30-fold higher than that of the parental strain, reaching 33.6 mg/L (Fig. 5; Table 1). This result indicated that the implementation of the NPP synthase orthogonal pathway is an adequate strategy for the channeling of metabolic flux towards limonene production in *A. gossypii*. Accordingly, the utilization of the NPP synthase was chosen to further optimize the MVA pathway.

### Transcriptional analysis and engineering the MVA pathway in *A. gossypii*

**Fig. 6:**
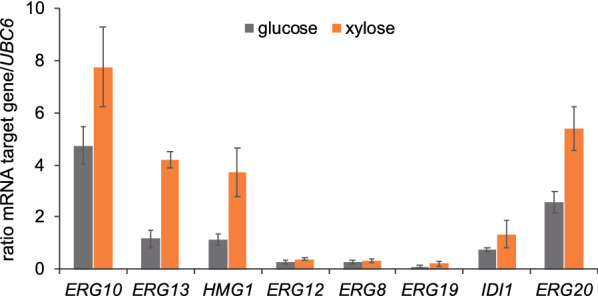
Transcriptional analysis of the mevalonate pathway. Quantitative real-time PCR of the genes controlling the mevalonate pathway in *A. gossypii*. A xylose-utilizing strain containing the overexpression of the XR-XDH-XK pathway was used. Total RNA was obtained from cultures grown for 48 h in MA2 media either with glucose or xylose as the only carbon source. Transcription levels of the genes were normalized using the *A. gossypii UBC6* gene as a reference. The results are the means of two independent experiments performed in duplicate and are expressed as a ratio of the cDNA abundances of the target genes with respect to the *UBC6* mRNA levels

**Fig. 7:**
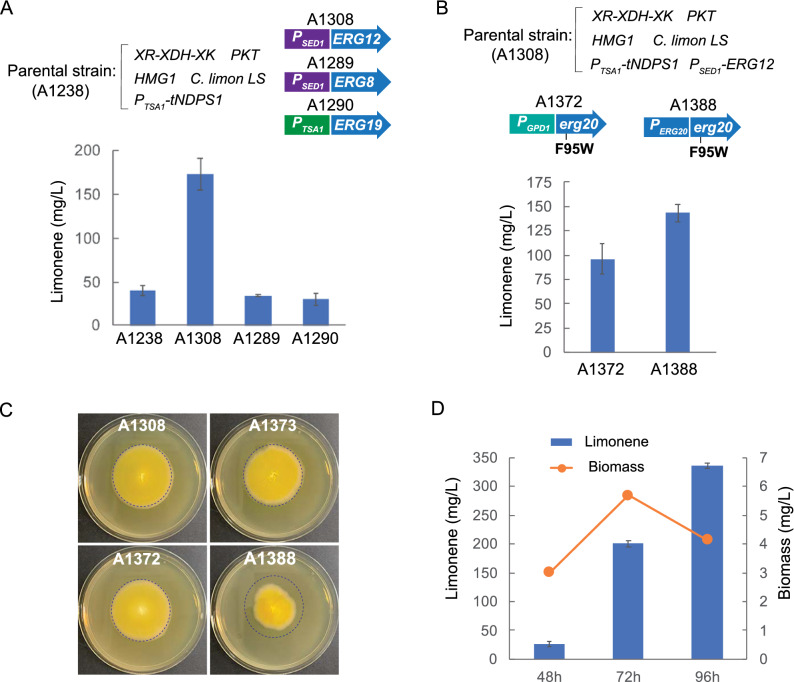
Optimization of the production of limonene in *A. gossypii*. **A** The genes *ERG12*, *ERG8* and *ERG19* were overexpressed using the specified strong promoters (*P*_SED1_ or *P*_TSA1_). The parental strain was A1238. **B** The allele *erg20*^*F95W*^ was combined with the *ERG12* overexpression in the A1308 strain. The mutant *erg20*^*F95W*^ was expressed either under a strong promoter (*P*_GPD1_*-erg20*^*F95W*^) or from its native promoter (*erg20*^*F95W*^). **C** Growth phenotype of engineered strains expressing different alleles of *ERG20* in the A1308 parental strain. A1373 contains *P*_GPD1_*-ERG20*; A1372 contains *P*_GPD1_*-erg20*^*F95W*^; A1388 contains *P*_ERG20_*-erg20*^*F95W*^. The blue circle represents the growth of the wild-type *ERG20*. Solid MA2 plates were cultured for 48 h. **D** The strain A1308 was cultured at the indicated time points. Limonene titers and biomass (dry cell weight) were calculated. The production of limonene in the engineered strains was measured in flask cultures grown for 72 h (except for D) with 0.5% glucose plus 2% xylose as the carbon sources. The results are the means of two independent experiments performed in duplicate

The regulation of gene transcription often determines the metabolic flux dynamics through cellular pathways. Hence, we carried out a transcriptional analysis of the genes controlling the MVA pathway in *A. gossypii*. A xylose-utilizing strain that overexpresses the XR-XDH-XK pathway was cultured both in glucose and xylose-based media for 48 h when the carbon source is still not depleted and the mRNA integrity is not affected by the mycelial lysis. Total mRNA was obtained and the transcription of the MVA pathway was evaluated by qPCR. We found that most of the genes from the MVA pathway showed a higher level of transcription when xylose was used as the carbon source (Fig. 6). In particular, *ERG10*, *ERG13*, *HMG1* and *ERG20* were strongly induced by xylose. However, in contrast, *ERG12*, *ERG8* and *ERG19* showed an extremely low level of transcription in both conditions, far below the transcriptional level of the housekeeping gene *UBC6* (Fig. 6). Accordingly, the overexpression of *ERG12*, *ERG8* and *ERG19* was assessed in combination with the utilization of the NPP synthase pathway. Both *ERG12* and *ERG8* were overexpressed using the strong promoter *P*_SED1_, while the overexpression of *ERG19* was carried out using the strong promoter *P*_TSA1_ [15]. The engineered strains were cultured in xylose-based media and the limonene production was measured. While the overexpression of both *ERG8* and *ERG19* did not result in an increased limonene titer, the overexpression of *ERG12* triggered a substantial improvement in its production, reaching 173.3 mg/L of limonene after 72 h in flasks cultures (Fig. 7A; Table 1). This titer represents about a 125-fold increase over the initial production measured in the strain that overexpressed the PKT pathway, the endogenous *HMG1* gene and the *C. limon tLS*.

We also checked the result of combining *ERG12* overexpression with the effect of the *erg20*^*F95W*^ allele. Unexpectedly, the limonene titer was reduced to 96 mg/L (Fig. 7B; Table 1), thus indicating the overexpression of the NPP synthase pathway in the presence of the *erg20*^*F95W*^ mutation is detrimental to limonene production. In this regard, an engineered strain expressing the *erg20*^*F95W*^ allele under its native promoter (i.e. not overexpressed) showed a higher limonene titer (143.3 mg/L) compared with the titer obtained when *erg20*^*F95W*^ was overexpressed (Fig. 7B; Table 1); however, the growth of that strain was partially compromised (Fig. 7C).

The growth phase can also influence the productivity of limonene in the *A. gossypii* cultures. Therefore, we analyzed the production of limonene in the aforementioned best-performing strain (Fig. 7A) during the early (48 h), mid (72 h) and late (96 h) growth phases, using xylose-based media. Our results showed that, while the production of biomass was maximal at 72 h of culture, the production of limonene peaked at a later stage (96 h), when mycelial lysis was evident (Fig. 7D; Table 1). Moreover, the highest limonene titer using our best-performing engineered strain reached 336.4 mg/L, indicating that the manipulations of the mevalonate pathway presented here can increase the production of limonene up to 243-fold greater than that of the original strain.

## Discussion

Xylose is the second most abundant sugar in lignocellulosic feedstocks [24], and the utilization of xylose-rich carbon sources for bioproduction constitutes an important challenge for biotechnology. Xylose bioconversion has been proposed in different yeasts and fungal platforms for the production of value-added biofuels and chemicals. These bioproducts include sugar alcohols such as xylitol, pyruvate-derived chemicals such as lactate and acetyl-CoA-derived products such as biolipids or terpenes [24]. In this regard, *A. gossypii*, which is a flavinogenic industrial fungus, has been described as a potential microbial factory for the production of biolipids from xylose-rich hydrolysates [18, 20]. Accordingly, the xylose assimilation metabolism of *A. gossypii* can be redirected for the production of terpenes such as limonene.

In this work, we have developed engineered strains of *A. gossypii* that produce limonene from xylose-based culture media. A truncated form of the limonene synthase from *C. limon* (CltLS) was used, demonstrating a high specificity for the biosynthesis of limonene in *A. gossypii*, as previously described in other microorganisms [6, 37]. In this regard, the utilization of different LSs for limonene bioproduction has been reported [6]. In particular, two different LSs from *C. limon*, CltLS1 and CltLS2, have shown different substrate specificity for GPP and NPP, with CltLS1 capable of using both substrates [32]. Also, engineering of LSs from various species has been shown either to enhance the enzymatic activity or to modify the enzyme selectivity [4].

The synthesis of HMG-CoA is a rate-limiting step in the MVA pathway due to the regulation of Hmg1. The expression of a truncated tHmg1 enzyme lacking the N-terminal domain, which is involved in the feedback regulation of the enzyme, is a widely accepted strategy to overcome this bottleneck [6, 7]. However, our results show that the overexpression of the full-length Hmg1 provides higher limonene titers than the use of two different truncated isoforms. Similar results were reported for *Y. lipolytica* strains producing limonene and linalool [26–28], suggesting that the Hmg1 enzyme from *A. gossypii* and *Y. lipolytica* might exhibit uncommon regulatory features. In this regard, the overexpression of a truncated tHmg1 in *S. cerevisiae* did not improve the production of β-carotene in xylose-based culture media, although the expression of the native Hmg1 was significantly enhanced (more than twofold) [25], as we could also observe in *A. gossypii* for limonene production. Therefore, the utilization of xylose as a carbon source promotes the bioproduction of limonene. In addition, the PKT pathway can contribute to further increasing the supply of acetyl-CoA and, consequently, enhance the production of limonene, as we have also reported for the production of biolipids [20].

The utilization of IPP and DMAPP for the biosynthesis of prenylated intermediates (i. e. GPP, NPP, FPP, etc.) seems to be another metabolic bottleneck for the production of terpenes. The biosynthesis of both GPP and FPP, which are precursors of different terpenes, is catalyzed by a bifunctional enzyme encoded by *ERG20*. We found that the overexpression of *ERG20* caused a notable decrease in the limonene titer, suggesting that the metabolic flux from IPP/DMAPP was mostly channeled toward the biosynthesis of FPP and derivatives, instead of limonene. This result highlights the effect of the GPP/FPP ratio on limonene production. Accordingly, three main strategies have been proposed to avoid this metabolic bottleneck: (i) the overexpression of *IDI1* that contributes to the increase of the DMAPP pool and promotes the synthesis of GPP [29]; (ii) the utilization of *ERG20* mutant alleles with a reduced FPP synthase activity [29, 30], which generates a higher GPP/FPP ratio; and (iii) the utilization of synthetic orthogonal pathways based on NPP synthases that can bypass Erg20 [9, 32, 36]. In *A. gossypii*, the overexpression of *IDI1* led to a 64% increase in the limonene titer. The overexpression of a double mutant *erg20*^*F95W/N126W*^ resulted in a lethal phenotype in *A. gossypii*, suggesting that the FPP level in the mutant strain is not sufficient for the biosynthesis of essential sterols, as described for the deletion of the yeast *ERG20* [39]. Likewise, the overexpression of the single mutants also produced an increase in the limonene titer (69.6% and 110.1% higher than that of the parental strain, using the N126W and F95W mutants, respectively). In contrast, the implementation of an orthogonal pathway in *A. gossypii*, through the overexpression of an NPP synthase from *S. lycopersicum*, drastically improved the production of limonene to 2334.8%. This indicates that the metabolic flux was efficiently channeled toward NPP and, importantly, that the *C. limon* tLS was able to transform NPP into limonene in *A. gossypii*. The utilization of orthogonal pathways has been described for the production of limonene and sabinene in *Y. lipolytica* and *S. cerevisiae* [28, 32, 34]. A limitation of the use of NPP-based orthogonal pathways for the production of monoterpenes can involve the selectivity of monoterpene synthases for the utilization of either GPP or NPP. However, protein engineering of monoterpene synthases can help to change the efficiency and specificity of the enzymes to accept alternative substrates [4, 34].

Three genes (*ERG8*, *ERG12* and *ERG19*) of the mevalonate pathway are expressed below the level of the housekeeping gene *UBC6* in *A. gossypii* but only the overexpression of *ERG12* elicited an increase in the production of limonene. In this regard, both transcriptional and enzymatic regulation have been documented in different steps of the mevalonate pathway in yeast and other eukaryotes [40]. Indeed, the overexpression of *ERG12* has also been described previously to increase the production of monoterpenes in different organisms [6, 41]. In *A. gossypii*, the simultaneous overexpression of the native *ERG12* and the *tNDPS1* from *S. lycopersicum* provides a maximum limonene titer of 336.4 mg/L, which represents one of the highest titers obtained using a eukaryotic microbial system (Table 2).

**Table 2:** Limonene titer in engineered microorganisms

Organism	Carbon source; culture mode	Limonene synthase	Relevant modifications	Titer (mg/L)	Refs.
*E. coli*	Glycerol; bioreactor; fed-batch	*Mentha spicata*, codon-optimized, truncated	Plasmid for gene overexpression of: *atoB* and *idi* from *E. coli; ERG13, tHMG1, ERG12, ERG8* and *ERG19* from *S. cerevisiae;* and *tGPPS* from *Abies grandis*	3630	[43]
*E. coli*	Glucose; shake flask; fed-batch	*M. spicata*, codon-optimized	*MvaE* and *MvaSA110G* from *Enterococcus faecalis* with synthetic RBS; *MVK* (*ERG12*) from *Methanosarcina mazei*; *ERG8*, *ERG19* and *IDI1* from *S. cerevisiae*; and tNDPS1 from *S. lycopersicum*	1290	[44]
*E. coli*	Glycerol; bioreactor; fed-batch	*M. spicata*, codon-optimized, truncated	*atoB* from *E. coli; ERG13, tHMG1, ERG12, ERG8, ERG19* and *IDI1* from *S. cerevisiae*; and *tGPPS* from *A. grandis*	1350	[45]
*S. cerevisiae*	Glucose and ethanol; shake flask, fed-batch	*C. limon*, codon-optimized, truncated	*tHMG1*, *IDI1*, *tNDPS1* from *S. lycopersicum*; and *ERG20*^*F96W−N127W*^	917.7	[32]
*S. cerevisiae*	Galactose:raffinose (2:1); shake flask; semi-batch	*C. limon*, mutant H570Y	*HMG2*^*K6R*^, gene fusion *ERG20*^*N127W*^*-SlNDPS1*	166	[34]
*Y. lipolytica*	Glucose; shake flask	*A. rugosa*, codon-optimized, truncated	*HMG1*, *ERG12*, and *tNDPS1* from *S. lycopersicum*	23.56	[28]
*Y. lipolytica*	Glycerol and citrate; bioreactor; fed-batch	*Agastache rugosa*, codon-optimized, truncated	*HMG1*, *ERG12*, *tNDPS1* from *S. lycopersicum*	165.3	[46]
*Y. lipolytica*	Glucose; bioreactor; fed-batch	*C. limon* or *M. spicata*	*HMG1*. Optimization of fermentation conditions	D-limonene:11.7; L-limonene:11.08	[26]

The combination of the synthetic orthogonal pathway with the expression of the mutant allele *erg20*^*F95W*^ did not improve the limonene titer, although the expression of *erg20*^*F95W*^ from its native promoter affects the growth of the engineered strain, probably due to a reduced FPP availability for sterol biosynthesis. The growth defect is restored when *erg20*^*F95W*^ is overexpressed under a strong promoter; however, the increase of the FPP level contributes to the fall of the limonene titer in that strain. Thus, an adequate balance of the GPP/NPP/FPP levels must be preserved to produce the highest levels of limonene.

Additional optimization of the culture conditions would help to increase the limonene titer in *A. gossypii*. Indeed, the utilization of culture media containing mixed formulations of carbon sources (lignocellulosic hydrolysates, molasses or crude glycerol) is an effective strategy to enhance the production of biolipids in *A. gossypii* [18]. The engineered strains presented here can be readily used for the exploitation of xylose-rich residues and by-products. In this regard, the utilization of xylose in *A. gossypii* can be further optimized by overexpressing the *afl205c*^*N355V*^ allele, which improves the consumption of pentose sugars in culture media with mixed carbon sources [42].

## Conclusions

This study demonstrates the potential of engineered strains of *A. gossypii* as efficient biocatalysts for the production of limonene from xylose. The present work represents a proof of principle for the production of terpenes from xylose-rich feedstocks such as lignocellulosic hydrolysates. The utilization of functional heterologous terpene synthases together with the overexpression of the native *HMG1* gene enables the implementation of terpene production systems in *A. gossypii*. In addition, the optimization of the mevalonate pathway can be achieved through the overexpression of both the native *ERG12* and the heterologous *tNDPS1* from *S. lycopersicum*. Further optimization of the bioprocess yield can be carried out by improving the cultivation mode using fed-batch bioreactors. Also, the suitability of *A. gossypii* as a cell factory opens new opportunities for the production of high-value limonene derivatives such as α-terpineol or perillyl alcohol from xylose-rich substrates.

## Methods

### *Ashbya gossypii* strains and growth conditions

The *A. gossypii* ATCC 10,895 strain was used as the wild-type strain. The *A. gossypii* strains used in this study are listed in Additional file 4. *A. gossypii* flask liquid cultures were initiated with spores (10^6^ spores per liter) and carried out at 28 ºC in an orbital shaker at 200 rpm. For limonene production MA2 rich medium with 0.5% glucose plus 2% xylose as carbon source was used [20, 47]. The *A. gossypii* transformation methods, sporulation conditions and spore isolation were as previously described [47]. Concentrations of 250 mg/L for Geneticin (G418) (Gibco-BRL) were used where indicated.

*Gene targeting methods.* Transformation cassettes for genomic integration were used for the overexpression of either endogenous or heterologous genes. For the overexpression of *A. gossypii* endogenous genes, the transformation cassettes comprised the *loxP-KanMX-loxP* selectable marker, conferring resistance to G418, followed by the sequence of a strong promoter (*P*_GPD1_, *P*_SED1_ or *P*_TSA1_) [15]. The overexpression cassettes were PCR-amplified using specific primers for each gene (Additional file 5), providing recombinogenic flanks (75–100 bp) for the genomic integration of the cassettes by homologous recombination (Additional file 6). The overexpression cassettes were integrated upstream of the ATG initiator codon of each gene for the overexpression of the full-length isoforms. Alternatively, for the overexpression of truncated isoforms of HMG1, the cassettes were targeted to the selected sequence of the *HMG1* CDS and the cassettes comprised an ATG initiator codon after the promoter sequence.

For the overexpression of heterologous genes, the integrative cassettes were assembled using a Golden Gate method as previously described [14]. The integrative cassettes comprised recombinogenic flanks, a *loxP-KanMX-loxP* (G418) marker and the transcriptional unit for each gene overexpression (Additional file 6). The overexpression cassettes for the PKT pathway (i.e. *pta* from *Bacillus subtilis* and *xpkA* from *Aspergillus nidulans*) were previously described [20]. However, in this work a new overexpression cassette for the combined expression of both transcriptional units was assembled with recombinogenic flanks targeting the *AGL034C* locus. For the overexpression of the *C. limon LS* gene, recombinogenic flanks targeting the *AFR171W* locus were used, and the regulatory sequences were the strong promoter *P*_GPD1_ and the terminator *T*_PGK1_. For the overexpression of the *tNDPS1* gene from *S. lycopersicum*, recombinogenic flanks targeting the *ABR025C* locus were used, and the regulatory sequences were the strong promoter *P*_TSA1_ and the terminator *T*_ENO1_. The synthetic codon-optimized sequences of both *C. limon LS* and *S. lycopersicum tNDPS1*, lacking the plastid targeting signal, were obtained from Integrated DNA Technologies (USA) (Additional file 1).

Spores of *A. gossypii* were transformed with the corresponding integrative cassette, and primary heterokaryon clones were selected in G418-containing medium. Homokaryon clones were obtained after the sporulation of the primary transformants. The genomic integration of each overexpression cassette was verified by analytical PCR followed by DNA sequencing. Gene overexpression was confirmed by qRT-PCR (Additional file 7). The *loxP-KanMX-loxP* marker contained two *loxP* inverted sequences that enabled the selection marker to be eliminated and reused by expressing a Cre recombinase, as previously described [48].

### CRISPR/Cas9 editing of the *A. gossypii* ERG20 gene

The adapted CRISPR/Cas9 system for *A. gossypii* [17] was used for the genomic edition of *ERG20*. A new synthetic sgRNA-dDNA was designed to introduce two substitutions (F95W and N126W) in Erg20 (see Additional file 1 for sequences details). The synthetic sgRNA-dDNA was flanked by two *Bsa*I sites that, after digestion, generated 4-nucleotide sticky ends to facilitate its assembly in the CRISPR/Cas9 vector [17]. The resulting plasmid was used to transform *A. gossypii* spores and heterokaryotic clones were selected in G418-containing medium. Sporulation of the primary heterokaryotic transformants in G418-free medium prevented the genomic integration of the plasmid and enabled the isolation of homokaryotic clones. The presence of the designed mutations was confirmed by analytical PCR followed by DNA sequencing. The analytical PCR was carried out with a primer (ERG20-CRISPR-ver-R1) that exclusively anneals to edited genome templates (Fig. 4).

### Quantitative real-time PCR

A LightCycler 480 real-time PCR instrument (Roche) was used to perform quantitative real-time PCR (qPCR) experiments, using PowerUp™ SYBR™ Green Master Mix (Applied Biosystems), and following the manufacturer’s instructions. Liquid cultures with selected strains were carried out in 50 mL of MA2 medium for 48 h. Total RNA samples were obtained as described previously [49], and cDNA samples were prepared using the High-Capacity cDNA Reverse Transcription kit (Applied Biosystems). Primer sequences are listed in Additional File 5. All qPCR reactions were performed in duplicate, and in two independent experiments. Quantitative analyses were carried out using the LightCycler 480 software. The mRNA level of the target genes was normalized to that of the housekeeping gene *AgUBC6* and was calculated using the 2 − ∆∆Ct method.

### Limonene extraction for gas chromatography and mass spectrometry analyses (GC–MS)

Flask cultures for limonene production were initiated with 10^6^ spores in a total volume of 40 mL of MA2 medium (0.5% glucose plus 2% xylose as the carbon source) with a 5% dodecane overlay. The cultures were harvested after 72 h and centrifuged for 10 min at 4400 rpm. For limonene quantification, the upper dodecane phase was collected in Eppendorf tubes, centrifuged for 5 min at 13,000 rpm, placed in sealed glass vials and stored at – 80 ºC until use. For biomass quantification, the cultures without the dodecane layer were resuspended and mycelial biomass was collected by filtration using pre-weighed filter papers. The dry cell weight (DCW) was determined after drying the mycelial biomass at 50 ºC.

For GC–MS analysis, 10 µL of thawed dodecane samples were diluted 1/20 with ethyl acetate and 100 µL of each diluted sample were placed in GC–MS glass vials. GC–MS was carried out in an Agilent 7890A GC System and Agilent MS 220 Ion Trap GC/MS, using a VF 5MS column (30 m long, 0.25 mm internal diameter and 0.20 μm of film). For the analyses, helium was used as carrier gas at a flow rate of 1 mL/min with a split ratio of 20:1. The injector temperature was 270 °C and the interface temperature was 270 °C. The oven program was as follows: initial temperature of 50 °C for 5 min, a ramp of 70 °C/min to 270 °C and a final temperature of 270 °C for 5 min. Limonene, pinene and sabinene standards (Sigma-Aldrich) were used for quantification.

## Supplementary Information

**Additional file 1.** Synthetic DNA sequences used in this work. DNA sequences of heterologous genes and sgRNA-dDNA for CRISPR edition of *ERG20*. The genetic elements of the sgRNA-dDNA are indicated in different colors.

**Additional file 2. **CLUSTAL alignment of the Erg20 proteins from *A. gossypii* and *S. cerevisiae*. Identical residues are black; similar residues are blue; not similar residues are red.

**Additional file 3. **Sequencing of the *A. gossypii erg20* mutants. Sequencing chromatograms of the *A. gossypii erg20* mutants. The *erg20* heterokaryotic mutant contains both nuclei with *erg20*^*F95W-N126W*^ and *erg20*^*F95W*^.

**Additional file 4. ***A. gossypii* strains used in this study. List of *A. gossypii* strains used in this study.

**Additional file 5. **List of primers used in this study. List of primers used in this study.

**Additional file 6. **Diagrams of the overexpression strategies used in this study. Schematic representation of the integrative cassettes used for the overexpression of both endogenous and heterologous genes.

**Additional file 7.** Title of data: qPCR analysis of the different overexpression modules used in the study. Total RNA was obtained from cultures of the corresponding strain grown during 48h in MA2 media. Transcription levels of the genes were normalized using the *A. gossypii UBC6* gene as a reference. The results are the means of two independent experiments performed in duplicate and are expressed as a ratio of the cDNA abundances of the target genes with respect to the *UBC6* mRNA levels.

## Data Availability

Not applicable.
