# Supplementary material for: Metabolic engineering of Ashbya gossypii for limonene production from xylose
Source: Biotechnol Biofuels Bioprod. 2022 Jul 15;15:79. doi: 10.1186/s13068-022-02176-0 (PMC9284773; doi:10.1186/s13068-022-02176-0)
Supplement: Supplementary file 1 — Additional file 1. Synthetic DNA sequences used in this work. DNA sequences of heterologous genes and sgRNA-dDNA for CRISPR edition of ERG20. The genetic elements of the sgRNA-dDNA are indicated in different colors. [file 13068_2022_2176_MOESM1_ESM.pdf]

Additional File 1. Synthetic DNA sequences used in this work.

Gene name	Sequence (5'-3')
tLS –<br>*Limonene synthase* (*Citrus limon*)	ATGAGACGTAGCGCCAACTACCAGCCTTCTATCTGGGACCACGACTTCTTGCAGTCTTTGAAC<br>TCTAACTATACCGACGAGGCCTACAAGAGGAGAGCCGAGGAGTTGCGTGGTAAGGTGAAGA<br>TCGCCATCAAGGACGTGATCGAGCCATTGGACCAGTTGGAGTTGATCGACAACTTGCAGAGA<br>TTGGGCTTGGCTCACAGATTCGAGACGGAGATCCGCAACATCTTGAACAACATCTACAACAA<br>CAACAAGGACTACAACTGGAGAAAGGAGAACTTGTACGCCACCTCCTTGGAGTTCCGCTTGC<br>TAAGACAGCACGGCTACCCAGTGTCTCAGGAGGTCTTCAACGGCTTCAAGGACGACCAGGG<br>TGGTTTCATTTGCGACGACTTTAAGGGCATCTTGTCGTTGCACGAGGCCTCCTACTACAGCTT<br>GGAGGGTGAGTCCATCATGGAAGAGGCTTGGCAGTTCACCTCTAAGCACTTGAAGGAGGTTA<br>TGATCAGCAAGAACATGGAGGAGGACGTGTTCGTGGCCGAGCAGGCAAAGCGTGCCTTGGA<br>GTTGCCATTGCACTGGAAGGTCCCAATGTTGGAGGCCAGATGGTTCATCCACATCTACGAGA<br>GACGCGAGGACAAGAACCACTTGTTGTTGGAGTTGGCTAAGATGGAGTTCAACACGTTGCAG<br>GCTATCTACCAGGAGGAGTTGAAGGAGATTTCTGGCTGGTGGAAGGACACTGGTTTGGGTGA<br>GAAGTTGTCTTTCGCGAGAAACAGATTGGTCGCCTCGTTCTTGTGGTCTATGGGCATCGCTTT<br>CGAGCCACAGTTCGCCTACTGTAGACGCGTGTTGACGATCTCCATCGCCCTAATCACCGTTA<br>TCGACGACATCTACGACGTGTACGGTACTTTGGACGAGTTGGAGATCTTCACCGACGCCGTC<br>GAGAGATGGGACATCAACTACGCTCTCAAGCACTTGCCAGGCTACATGAAGATGTGCTTCTT<br>GGCCTTGTACAACTTCGTCAACGAGTTCGCCTACTACGTTTTGAAGCAACAGGACTTCGACTT<br>GTTGTTGTCGATCAAGAACGCTTGGTTGGGCTTGATCCAGGCCTACTTGGTGGAGGCCAAGT<br>GGTATCACTCGAAGTACACGCCAAAGTTGGAGGAGTACCTAGAGAACGGCTTGGTTTCCATC<br>ACCGGTCCATTGATCATCACCATCAGCTACTTGTCCGGTACTAACCCAATCATCAAGAAGGAG<br>TTGGAGTTTTTGGAGAGCAACCCAGACATCGTCCACTGGTCCTCTAAGATCTTCAGATTGCAG<br>GACGACTTGGGCACCAGCTCTGACGAGATCCAGCGCGGCGACGTTCCTAAGTCTATCCAGT<br>GTTACATGCACGAAACCGGCGCCTCCGAGGAGGTCGCTCGCCAGCACATCAAGGACATGAT<br>GCGTCAGATGTGGAAGAAGGTCAACGCCTACACCGCTGACAAGGACTCCCCATTGACCGGC<br>ACTACCACTGAGTTCCTATTGAACTTGGTGAGAATGAGCCACTTCATGTACTTGCACGGTGAC<br>GGCCACGGCGTTCAGAACCAGGAAACCATCGACGTGGGCTTCACCTTGTTGTTCCAGCCAAT<br>CCCATTGGAGGACAAGCACATGGCTTTCACCGCCTCTCCAGGTACTAAGGGCTAG
tNDPS1 – NPP synthase (Solanum lycopersicum)	ATGTCTGCACGCGGTCTTAACAAAATCTCATGTTCTTTGAACCTTCAGACCGAGAAGCTATGC<br>TACGAGGACAACGACAACGACTTGGACGAGGAGTTGATGCCAAAGCACATCGCTTTGATCAT<br>GGACGGCAACAGAAGATGGGCCAAGGACAAGGGTTTGGAGGTGTACGAGGGCCACAAGCA<br>CATCATCCCAAAGTTGAAGGAGATCTGCGACATCTCCTCTAAGTTGGGTATCCAGATCATCAC<br>CGCCTTCGCCTTCTCTACCGAGAACTGGAAGAGATCGAAGGAGGAGGTGGACTTCCTATTGC<br>AGATGTTCGAGGAGATCTACGACGAGTTTTCTCGTTCTGGCGTCAGAGTGTCTATCATCGGCT<br>GCAAGTCCGACTTGCCAATGACCTTGCAGAAGTGTATCGCCTTGACCGAGGAAACCACCAAG<br>GGCAACAAGGGTCTGCACTTGGTCATCGCCCTCAACTACGGCGGCTACTACGACATCCTCCA<br>GGCCACCAAGAGCATTGTGAACAAGGCTATGAACGGCTTGTTGGACGTGGAGGACATCAACA<br>AGAACTTGTTCGACCAGGAGTTGGAGTCTAAGTGTCCAAACCCAGACTTGTTGATCAGAACC<br>GGTGGTGAGCAGAGAGTGTCCAACTTCTTGTTGTGGCAGTTGGCTTACACCGAGTTCTACTT<br>CACTAACACCCTCTTCCCAGACTTCGGCGAGGAGGACCTCAAGGAGGCTATCATGAACTTCC<br>AGCAGCGCCACAGACGCTTCGGCGGCCACACCTACTGA
sgRNA-dDNA erg20$^{F95W\text{-}N126W}$<br>***Bold: BsaI sites***<br>*Red: gRNA target*<br>*Blue: gRNA – Cas9 binding*<br>*Green: donor DNA*	CGGATCCCCGGGTTAATTAA**GGTCTC**ACATTTGTCGCCCACCTCCTCCACAGTTTTAGAGCTA<br>GAAATAGCAAGTTAAAATAAGGCTAGTCCGTTATCAACTTGAAAAAGTGGCACCGAGTCGGTG<br>GTGCACTTTTTGCAGGTCGACAGCGTAGCGCTCTTGGGGTGGTGCGTAGAGTTGTTGCAGG<br>CGTACTGGTTGGTGGCCGACGACATGATGGACAAGTCGATCACCCGGCGTGGTCAGCCCTG<br>CTGGTATCGAGTAGAAGAAGTTGGTGACATGGCCATCTGGGATGCTTTCATGCTGGAAGCGG<br>CCATCTACTGTTTGTTGAAGCGCAACTTCCGCGACCAGCCCTACTATGTGGACCTTCTCGAGT<br>TGTTGCACGACGTCACGTTCAGAGA**GAGACC**GTTTAAACGAGCTCGAATTC
