# Supplementary figures and images for: Metabolic engineering of Ashbya gossypii for limonene production from xylose
Source: Biotechnol Biofuels Bioprod. 2022 Jul 15;15:79. doi: 10.1186/s13068-022-02176-0 (PMC9284773; doi:10.1186/s13068-022-02176-0)

Additional file 2. CLUSTAL alignment of the Erg20 proteins from *A. gossypii* and *S. cerevisiae*

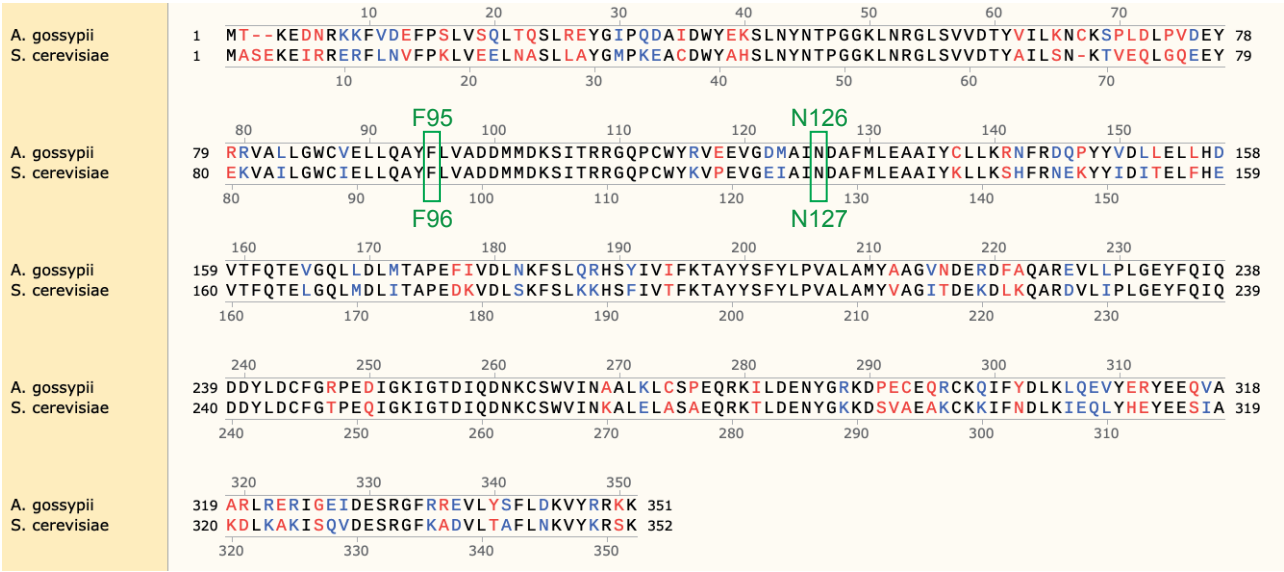

Supplement: Supplementary file 2 — Additional file 2. CLUSTAL alignment of the Erg20 proteins from A. gossypii and S. cerevisiae. Identical residues are black; similar residues are blue; not similar residues are red. [file 13068_2022_2176_MOESM2_ESM.pdf]

Overexpression of endogenous genes

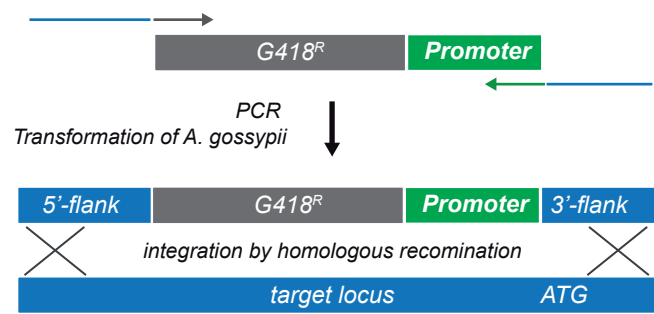

Overexpression of heterologous genes

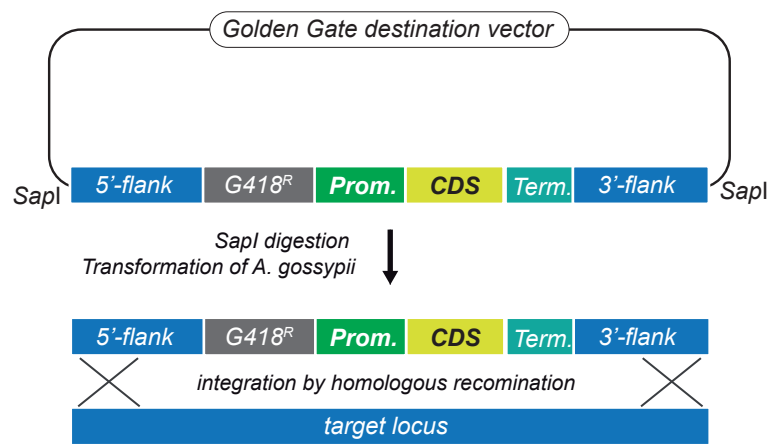

Supplement: Supplementary file 6 — Additional file 6. Diagrams of the overexpression strategies used in this study. Schematic representation of the integrative cassettes used for the overexpression of both endogenous and heterologous genes. [file 13068_2022_2176_MOESM6_ESM.pdf]

Additional file 7. qPCR analysis of the different overexpression modules used in the study

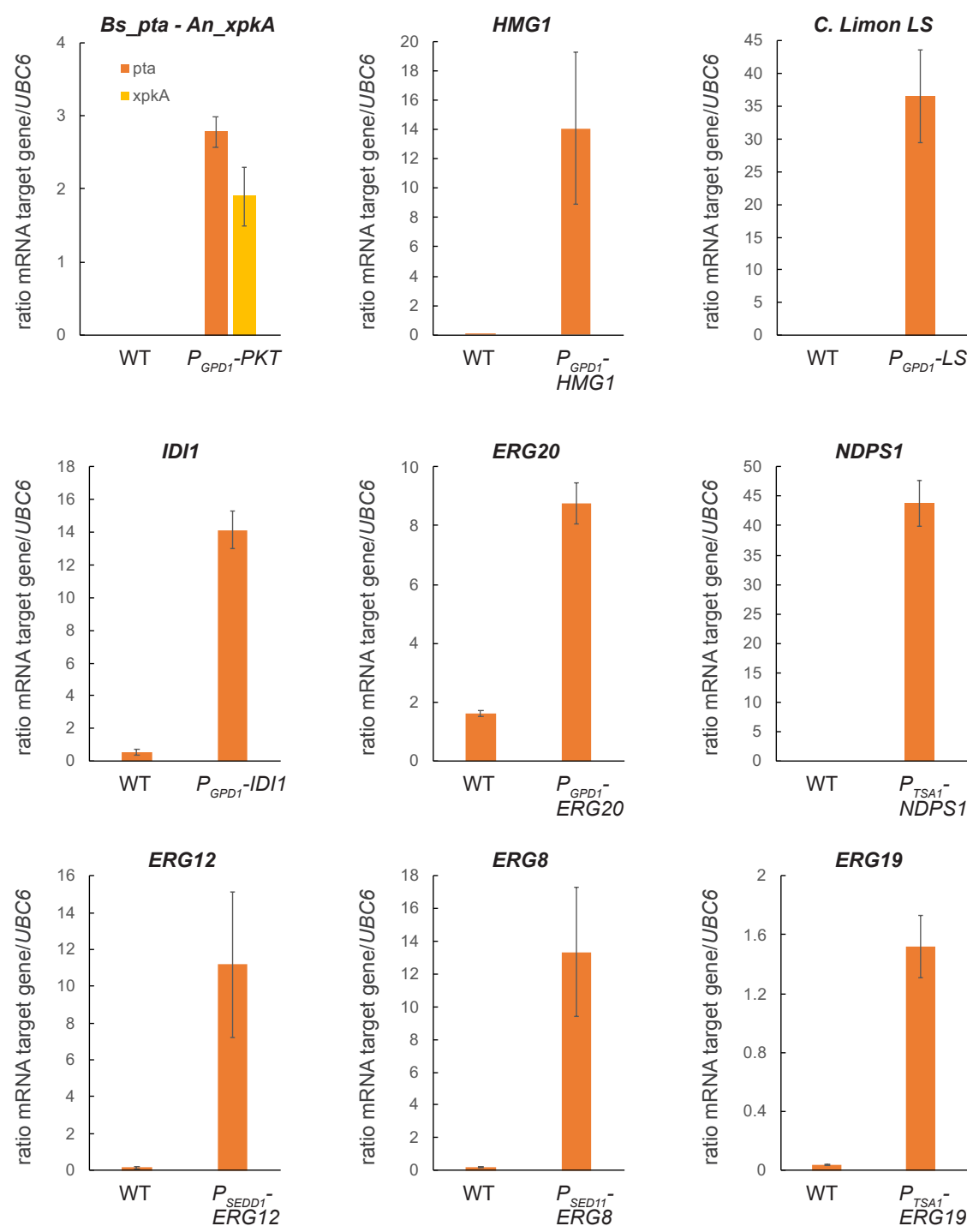

Supplement: Supplementary file 7 — Additional file 7. Title of data: qPCR analysis of the different overexpression modules used in the study. Total RNA was obtained from cultures of the corresponding strain grown during 48h in MA2 media. Transcription levels of the genes were normalized using the A. gossypii UBC6 gene as a reference. The results are the means of two independent experiments performed in duplicate and are expressed as a ratio of the cDNA abundances of the target genes with respect to the UBC6 mRNA levels. [file 13068_2022_2176_MOESM7_ESM.pdf]
