# Supplementary material for: Metabolic engineering of Ashbya gossypii for limonene production from xylose
Source: Biotechnol Biofuels Bioprod. 2022 Jul 15;15:79. doi: 10.1186/s13068-022-02176-0 (PMC9284773; doi:10.1186/s13068-022-02176-0)

Additional file 3. Sequencing of the *A. gossypii erg20* mutants

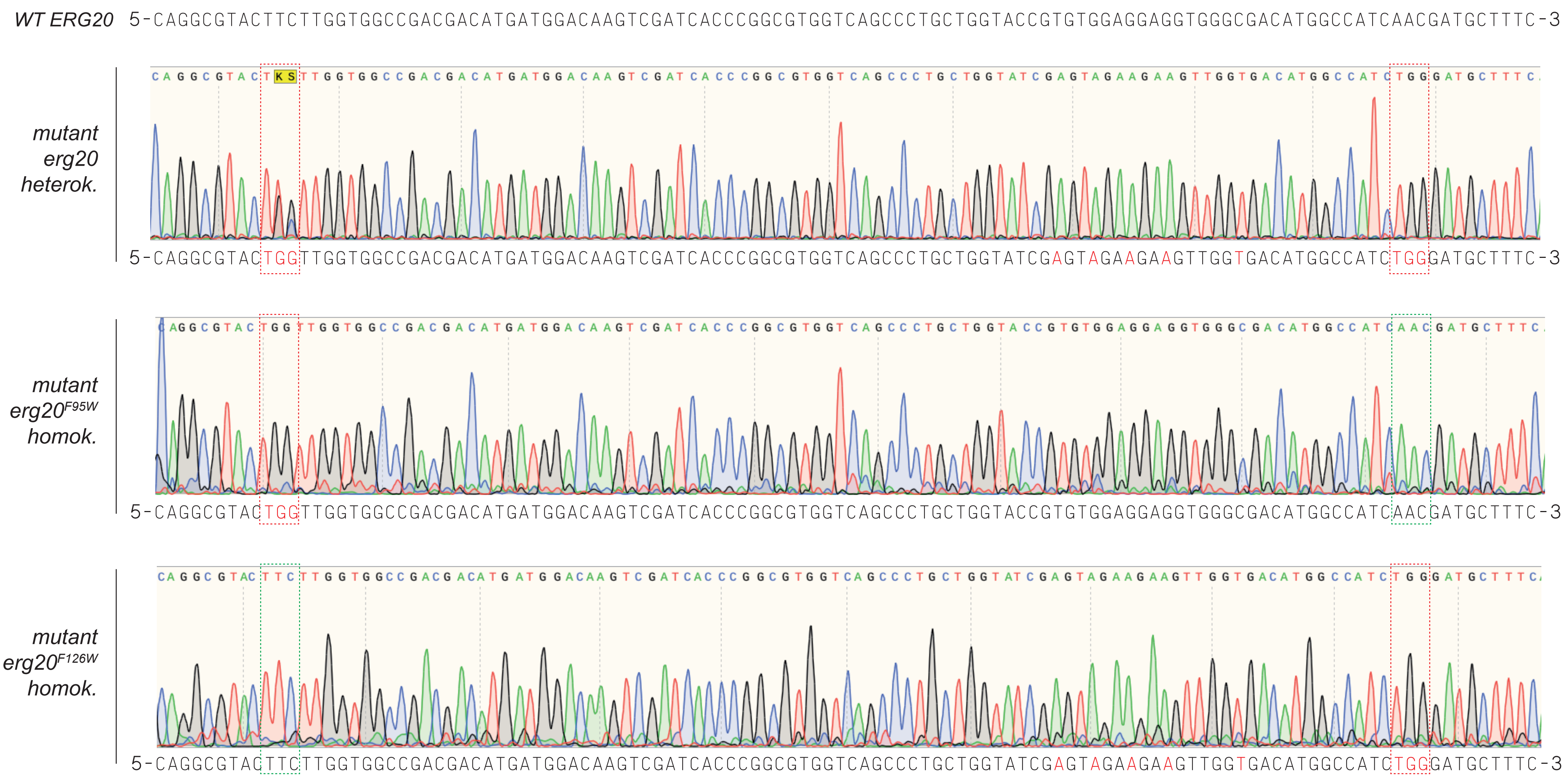

Supplement: Supplementary file 3 — Additional file 3. Sequencing of the A. gossypii erg20 mutants. Sequencing chromatograms of the A. gossypii erg20 mutants. The erg20 heterokaryotic mutant contains both nuclei with erg20F95W-N126W and erg20F95W. [file 13068_2022_2176_MOESM3_ESM.pdf]
