# Supplementary material for: Metabolic engineering of Ashbya gossypii for limonene production from xylose
Source: Biotechnol Biofuels Bioprod. 2022 Jul 15;15:79. doi: 10.1186/s13068-022-02176-0 (PMC9284773; doi:10.1186/s13068-022-02176-0)
Supplement: Supplementary file 4 — Additional file 4. A. gossypii strains used in this study. List of A. gossypii strains used in this study. [file 13068_2022_2176_MOESM4_ESM.pdf]

Additional File 4. *A. gossypii* strains used in this study.

Strain	Genotype	Source
WT	Wild type (ATCC 10895)	our lab stock
A665 (GXX)	P_{GPD1}-*GRE3*; P_{GPD1}-*XKS1*; P_{GPD1}-*XYL2*	our lab stock
A1117	*GXX*; P_{GPD1}-*tHMG1-2*; *afr171w*::P_{GPD1}-*C. limon LS*-T_{PGK1}	this work
A1123	*GXX*; P_{GPD1}-*HMG1*; *afr171w*::P_{GPD1}-*C. limon LS*-T_{PGK1}	this work
A1137	*GXX*; P_{GPD1}-*tHMG1-1*; *afr171w*::P_{GPD1}-*C. limon LS*-T_{PGK1}	this work
A1152	*GXX*; P_{GPD1}-*tHMG1-1*; *afr171w*::P_{GPD1}-*C. limon LS*-T_{PGK1}; *agl034c*::P_{GPD1}-*pta*-T_{PGK1} - P_{GPD1}-*xpkA*-T_{ENO1}	this work
A1171	*GXX*; P_{GPD1}-*tHMG1-2*; *afr171w*::P_{GPD1}-*C. limon LS*-T_{PGK1}; *agl034c*::P_{GPD1}-*pta*-T_{PGK1} - P_{GPD1}-*xpkA*-T_{ENO1}	this work
A1206	*GXX*; P_{GPD1}-*HMG1*; *afr171w*::P_{GPD1}-*C. limon LS*-T_{PGK1}; *agl034c*::P_{GPD1}-*pta*-T_{PGK1} - P_{GPD1}-*xpkA*-T_{ENO1}	this work
A1224	*GXX*; P_{GPD1}-*HMG1*; *afr171w*::P_{GPD1}-*C. limon LS*-T_{PGK1}; *agl034c*::P_{GPD1}-*pta*-T_{PGK1} - P_{GPD1}-*xpkA*-T_{ENO1}; P_{GPD1}-*IDI1*	this work
A1238	*GXX*; P_{GPD1}-*HMG1*; *afr171w*::P_{GPD1}-*C. limon LS*-T_{PGK1}; *agl034c*::P_{GPD1}-*pta*-T_{PGK1} - P_{GPD1}-*xpkA*-T_{ENO1}; *abr025c*::P_{TSA1}-*tNDPS1*-T_{ENO1}	this work
A1242	*GXX*; P_{GPD1}-*HMG1*; *afr171w*::P_{GPD1}-*C. limon LS*-T_{PGK1}; *agl034c*::P_{GPD1}-*pta*-T_{PGK1} - P_{GPD1}-*xpkA*-T_{ENO1}; P_{GPD1}-*ERG20*	this work
A1251	*GXX*; P_{GPD1}-*HMG1*; *afr171w*::P_{GPD1}-*C. limon LS*-T_{PGK1}; *agl034c*::P_{GPD1}-*pta*-T_{PGK1} - P_{GPD1}-*xpkA*-T_{ENO1}; P_{GPD1}-*ERG20*$^{(N126W)}$	this work
A1289	*GXX*; P_{GPD1}-*HMG1*; *afr171w*::P_{GPD1}-*C. limon LS*-T_{PGK1}; *agl034c*::P_{GPD1}-*pta*-T_{PGK1} - P_{GPD1}-*xpkA*-T_{ENO1}; *abr025c*::P_{TSA1}-*tNDPS1*-T_{ENO1}; P_{SED1}-*ERG8*	this work
A1290	*GXX*; P_{GPD1}-*HMG1*; *afr171w*::P_{GPD1}-*C. limon LS*-T_{PGK1}; *agl034c*::P_{GPD1}-*pta*-T_{PGK1} - P_{GPD1}-*xpkA*-T_{ENO1}; *abr025c*::P_{TSA1}-*tNDPS1*-T_{ENO1}; P_{TSA1}-*ERG19*	this work
A1304	*GXX*; P_{GPD1}-*HMG1*; *afr171w*::P_{GPD1}-*C. limon LS*-T_{PGK1}; *agl034c*::P_{GPD1}-*pta*-T_{PGK1} - P_{GPD1}-*xpkA*-T_{ENO1}; P_{GPD1}-*ERG20*$^{(F95W)}$	this work
A1308	*GXX*; P_{GPD1}-*HMG1*; *afr171w*::P_{GPD1}-*C. limon LS*-T_{PGK1}; *agl034c*::P_{GPD1}-*pta*-T_{PGK1} - P_{GPD1}-*xpkA*-T_{ENO1}; *abr025c*::P_{TSA1}-*tNDPS1*-T_{ENO1}; P_{SED1}-*ERG12*	this work
A1372	*GXX*; P_{GPD1}-*HMG1*; *afr171w*::P_{GPD1}-*C. limon LS*-T_{PGK1}; *agl034c*::P_{GPD1}-*pta*-T_{PGK1} - P_{GPD1}-*xpkA*-T_{ENO1}; *abr025c*::P_{TSA1}-*tNDPS1*-T_{ENO1}; P_{SED1}-*ERG12*; P_{GPD1}-*ERG20*$^{(F95W)}$	this work
A1273	*GXX*; P_{GPD1}-*HMG1*; *afr171w*::P_{GPD1}-*C. limon LS*-T_{PGK1}; *agl034c*::P_{GPD1}-*pta*-T_{PGK1} - P_{GPD1}-*xpkA*-T_{ENO1}; *abr025c*::P_{TSA1}-*tNDPS1*-T_{ENO1}; P_{SED1}-*ERG12*; P_{GPD1}-*ERG20*	this work
A1388	*GXX*; P_{GPD1}-*HMG1*; *afr171w*::P_{GPD1}-*C. limon LS*-T_{PGK1}; *agl034c*::P_{GPD1}-*pta*-T_{PGK1} - P_{GPD1}-*xpkA*-T_{ENO1}; *abr025c*::P_{TSA1}-*tNDPS1*-T_{ENO1}; P_{SED1}-*ERG12*; *ERG20*$^{(F95W)}$	this work
