# Supplementary material for: Metabolic engineering of Ashbya gossypii for limonene production from xylose
Source: Biotechnol Biofuels Bioprod. 2022 Jul 15;15:79. doi: 10.1186/s13068-022-02176-0 (PMC9284773; doi:10.1186/s13068-022-02176-0)
Supplement: Supplementary file 5 — Additional file 5. List of primers used in this study. List of primers used in this study. [file 13068_2022_2176_MOESM5_ESM.pdf]

Additional File 5. List of primers used in this study.

Primer	Sequence (5’-3’)	Purpose
HMG1-ins5	CAGGTTGACCGACTCTAAGGATATTGCGTGTATCAAGAGGATCTCAGTTGGC AGTGCGTTTGAGCG	*HMG1* overexpression
HIMG1-ins3	ATCGGGTGCTTCGCACAGAAGTAGGCGCTTAGCGCGAACGGCGTCACCACA TGCTTGATCTGCTTG	*HMG1* overexpression
HMG1-T1-ins3	GCGGGGACGACAATCGGAGTCGACCCTACCGAAAAGGAGGCCGTTTTCCGG GCTTTCTTTTTGTTG	*tHMG1-1* overexpression
HMG1-T2-ins3	ACCACCTCTTTGTTCTTCAACGTTTTCACTGCACCCTTCCGCAAAACGTCAAT CAAGGTCGCTAGCGGG	*tHMG1-2* overexpression
IDI1-ins5	CGGCTGCGATGGCAGCCCGGCGCTCGCCGCACTGGAAAAAGATATAACAGC GGGACGGCGGGTTTCGTTCTCGCAAGACAGGGTAATATAGATCTGGTCG	*IDI1* overexpression
IDI1-ins3	TTCACCAGTACCGCGTACGGCGACATACGCTCCTTGCCCACCCGCTGCATCA CCACCTTGCCTTGAACTACAACCGACATTGTGCGGTGTGTATGTGTGG	*IDI1* overexpression
P_{SED1}-ERG8-ins5	TGGTTATAATTATCTATTAATTGATATCAATTCATACGGTTTATATTCAATATAT TCCCTAGTACTCAA	*ERG8* overexpression
P_{SED1}-ERG8-ins3	AGTTGGGGTCAAGTACCAAATAGCCCCCAACTAGAAGAGCCTTACCTGGCGC ACTGAAAGCCCTTA	*ERG8* overexpression
P_{SED1}-ERG12-ins5	CCGCTAGACGTGAACCGCGGGCAGCGGCGCCATTGGTGGAATGCGGATGTC GCGGCCTCATTTTGC	*ERG12* overexpression
P_{SED1}-ERG12-ins3	TGATCACCTTCCCAGGCGCCGAAGTGATGAAGGGTAGCAACTCGCCTGTGC AAATGACTGTCATCG	*ERG12* overexpression
P_{TSA1}-ERG19-ins5	CGTCTGGCAGATCCCACTACACCGCAGGGTGCACCTATAGTGGCAAAAAATT TCCAGCGTTACGCACCTATACCACGCCACGGATCCCCGGGTTAATTAA	*ERG19* overexpression
P_{TSA1}-ERG19-ins3	CAGAGGCCCGCACACGCACAGGGACATACTGCGATGTTCACAGGCGCCGTT GTAGACGCAACGTAG	*ERG19* overexpression
ERG20-ins5	ATCGCAAAGTCAGCTCAAGTTCAGCTAGCTAGAATTCGACTTGGAGATAGTG GGTGTCAGTACGCTGTCAGAAGTATACAGGGTAATATAGATCTGGTCG	*ERG20* overexpression
ERG20-ins3	TCGCGCAAGGACTGCGTCAACTGTGACACCAACGATGGGAACTCGTCCACTA ACTTCTTCCTATTG	*ERG20* overexpression
kanB	CTGCAGCGAGGAGCCGTAAT	analytical PCR
AGL034C-a	CAGCTCATCACAACGCTTATCCA	analytical PCR
AFR171W-a	GATCGCTACGAAGACGAGAG	analytical PCR
ABR025CL-a	CCTAGCTGCTGTTGCGCTTA	analytical PCR
HMG1-a	CTTCGACGTCAAGCTCGTCT	analytical PCR
HMG1-b	GCGTCGAACTCCACCTTCAT	analytical PCR
IDI1-a	AGCTTCTGCTTGCTCTCCTG	analytical PCR
ERG8-a	GAACAGAAGAGACGCGGGAA	analytical PCR

ERG12-a	CAAGGGCTGTCTCGTTGACT	analytical PCR
ERG19-a	TGCTTTTATTCGCGACACACG	analytical PCR
ERG20-a	TGGTCAAGACAGACGAGCAC	analytical PCR
ERG20-CRISPR-ver-R1	TGTCACCAACTTCTTCTACTCGA	analytical PCR
ERG20-CRISPR-ver-rv	CTCGCACTCCGGGTCCTTAC	analytical PCR
ERG20-verF95-fw	GAGTTGTTGCAGGCGTACTGG	analytical PCR
UBC6-qPCR-fw	TCCGCCCGCGATCAGGATG	qRT-PCR
UBC6-qPCR-rv	CTTGCGCTTCGTGGAGTCCGTAGA	qRT-PCR
HMG1-qPCR-fw	CTCCAAGGGCGTCGAGTTCG	qRT-PCR
HMG1-qPCR-rv	GGCATGGAGACGGAGACGCG	qRT-PCR
ERG8-qPCR-fw	CACAGGTCGCGCATTGCCAA	qRT-PCR
ERG8-qPCR-rv	GTCCGCGCCGGATTCAGATG	qRT-PCR
ERG10-qPCR-fw	CGATCACGCGTGAGGAGCAG	qRT-PCR
ERG10-qPCR-rv	GTCTGCTGGCTCGTGGGCAG	qRT-PCR
ERG12-qPCR-fw	CGGTGGTACGGGCCTTCTGC	qRT-PCR
ERG12-qPCR-rv	GTAGAGCCGTCCGGCTGACG	qRT-PCR
ERG13-qPCR-fw	GCGTGGCTCGTACATGGAGC	qRT-PCR
ERG13-qPCR-rv	GGATGGCTCAACACGCGCTT	qRT-PCR
ERG19-qPCR-fw	GCAGCAGTGTCTGGCGGACC	qRT-PCR
ERG19-qPCR-rv	CGTCTGCTGCATACCGCTCG	qRT-PCR
ERG20-qPCR-fw	CAAGTCGCCGCTCGACTTGC	qRT-PCR
ERG20-qPCR-rv	CCAAGGCGACGGGCAAGTAGA	qRT-PCR
IDI1-qPCR-fw	CGGGGCACGATGAGGAGCAGA	qRT-PCR
IDI1-qPCR-rv	GGCGCCATGTAGTGGATACGGT	qRT-PCR
LSCl-qPCR-fw	CGCTTGGTTGGGCTTGATCC	qRT-PCR
LSCl-qPCR-rv	ATCTCGTCAGAGCTGGTGCC	qRT-PCR
tNDPS1-qPCR-fw	GGAGGTTGTACGAGGGCCACA	qRT-PCR
tNDPS1-qPCR-rv	CCTCCACGTCCAACAAGCCG	qRT-PCR
